# Efficacy of faecal microbiota transplantation in Crohn’s disease: a new target treatment?

**DOI:** 10.1111/1751-7915.13536

**Published:** 2020-01-20

**Authors:** Liyuan Xiang, Xiao Ding, Qianqian Li, Xia Wu, Min Dai, Chuyan Long, Zhi He, Bota Cui, Faming Zhang

**Affiliations:** ^1^ Medical Center for Digestive Diseases the Second Affiliated Hospital of Nanjing Medical University Nanjing 210011 China; ^2^ Key Lab of Holistic Integrative Enterology Nanjing Medical University Nanjing 211100 China; ^3^ Division of Microbiotherapy Sir Run Run Shaw Hospital Nanjing Medical University Nanjing 211166 China; ^4^ National Clinical Research Center for Digestive Diseases Xi'an 710032 China

## Abstract

The efficacy of faecal microbiota transplantation (FMT) in Crohn’s disease (CD) remains unclear due to lack of data. This study aimed to assess the value of FMT in treating CD‐related clinical targets. The use of FMT for CD as a registered trial (NCT01793831) was performed between October 2012 and December 2017. Seven therapeutic targets included abdominal pain, diarrhoea, hematochezia, fever, steroid‐dependence, enterocutaneous fistula and active perianal fistula. Each target was recorded as 1 (yes) or 0 (no) during the long‐term follow‐up for each patient. The primary outcome was the rate of improvement in each therapeutic target. Overall, 174 patients completed the follow‐up. The median follow‐up duration was 43 (interquartile range, 28–59) months. The median score of the total targets was 2 (range, 1–4) before FMT, and it decreased significantly at 1, 3, 6, 12, 24 and 36 months after FMT (*P* < 0.001 respectively). At 1 month after FMT, 72.7% (101/139), 61.6% (90/146), 76% (19/25) and 70.6% (12/17) of patients achieved improvement in abdominal pain, diarrhoea, hematochezia and fever respectively. Furthermore, 50% (10/20) of steroid‐dependent patients achieved steroid‐free remission after FMT. The present findings indicate that it is important to understand the efficacy of FMT in CD as a targeted therapy, especially for abdominal pain, hematochezia, fever and diarrhoea.

## Graphical abstract

This study aimed to assess the value of FMT in treating CD‐related clinical targets. The present findings indicate that it is important to understand the efficacy of FMT in CD as a targeted therapy, especially for abdominal pain, hematochezia, fever, and diarrhea.

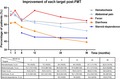

## Introduction

Crohn’s disease (CD) is a chronic inflammatory disease that is considered to result from a complex interplay between genetic susceptibility, environmental factors and altered gut microbiota. The therapeutic potential of faecal microbiota transplantation (FMT) in CD has recently been demonstrated (Gordon and Harbord, 2014; Kao *et al.*, 2014; Suskind *et al.*, 2015; Cui *et al*., 2015a; Vaughn *et al.*, 2016; Gutin *et al.*, 2019). In our pilot study between 2012 and 2014, 86.7% and 76.7% of patients with refractory CD achieved clinical improvement and remission at 1 month after a single FMT through mid‐gut respectively (Cui *et al*., 2015a). It was the first report of significant long‐term relief in abdominal pain in patients with CD following FMT (Cui *et al*., 2015a). In a subsequent study, we demonstrated that multiple fresh FMTs were effective in inducing and maintaining clinical remission in CD patients with intra‐abdominal inflammatory masses (He *et al.*, 2017). A subsequent pilot study explored the timing for the second course of FMT in order to maintain the clinical response of the first FMT. Based on the evaluation of 69 patients with CD, 3 months after the first FMT was recommended as the appropriate time for the second course of FMT in patients with CD (Li *et al.*, 2019).

However, the treatment for CD is generally individualized and patients might receive more than one line of therapy. The integrative treatment—called step‐up FMT strategy—consists of single or multiple FMTs combined with steroids, immunomodulators, and exclusive enteral nutrition (EEN) and has been applied to our clinical practice (Cui *et al*., 2015b; Cui *et al.*, 2016; Ding *et al.*, 2019). Based on our clinical observations, we believed that FMT may be beneficial in CD in many aspects which could be defined as therapeutic targets. Importantly, FMT is not similar to treatments that use pharmacological chemicals or antibodies; it is a novel therapy that is based on host‐microbial interactions. FMT may have more than one target in a host, such as controlling infection, relieving pain, managing chronic inflammation, stopping diarrhoea, maintaining haemostasis and as an aid in tapering off steroids. We hypothesized that assessing these responses following FMT can be useful in making clinical decisions on the use of FMT in the management of CD. Therefore, to test our hypothesis, we outlined serial clinical targets in this study to evaluate the efficacy of FMT in patients with CD.

## Results

### Patients and FMT

Two hundred and fourteen CD patients with therapeutic targets underwent FMT between October 2012 and December 2017. Of them, 40 patients were excluded for the following reasons: lost to follow‐up (*n* = 22), change in the diagnosis during the follow‐up (*n* = 7), concomitant CDI (*n* = 2), stoma (*n* = 3), glycogen storage disease (*n* = 1), and undergoing endoscopic balloon dilation (*n* = 4) and perianal surgery (*n* = 1) within two weeks before FMT. Finally, 174 patients were included in the analyses. The median follow‐up duration was 43 months (interquartile range, IQR: 28–59). The baseline characteristics of patients are presented in Table 1. FMT‐related data, such as donor, preparation, status and delivery route, are summarized in Table S1. Only one patient underwent FMT through colonic transendoscopic enteral tubing (TET) (Peng *et al.*, 2016) because of severe colonic lesions detected during colonoscopy and TET was inserted for infusion of mesalazine in the whole colon. Others received FMT through the mid‐gut, including endoscopy, nasojejunal tube and mid‐gut TET (Long *et al.*, 2018).

**Table 1:** Patient characteristics.

Items	Results
Total number	174
Sex, male, % (*n*)	68.4 (119)
Follow‐up duration, month (median; IQR)	43 (28–59)
Age at FMT, year (median; IQR)	33 (23–43)
Age at onset, year (median; IQR)	25 (18–36)
Age at diagnosis, year (median; IQR)	27 (20–37)
Time from diagnosis to first FMT, year (median; IQR)	3 (1–6)
Duration of disease, year (median; IQR)	5 (2–9)
Age at diagnosis, % (*n*)	
A1 (age < 17 years)	15.5 (27)
A2 (age between 17 and 40 years)	63.2 (110)
A3 (age > 40 years)	21.3 (37)
Location, % (*n*)	
L1 (ileal)	16.7 (29)
L2 (colonic)	16.7 (29)
L3 (ileocolonic)	58.0 (101)
L4 (upper gastrointestinal tract) ± (L1‐L3)	8.6 (15)
Behaviour, % (*n*)	
B1 (non‐stricturing, non‐penetrating)	43.7 (76)
B2 (stricturing)	39.1 (68)
B3 (penetrating)	17.2 (30)
Perianal disease	16.7 (29)
HBI (median; IQR)	8 (6–10)
HS‐CRP, mg l^−1^ (median; IQR)	21 (7–50)
Haemoglobin, g l^−1^ (median; IQR)	114 (100–132)
Albumin, g l^−1^ (median; IQR)	37 (31–41)
BMI, kg m^−2^ (median; IQR)	17.3 (16.0–19.5)
Treatment history	
5‐aminosalicylates, % (*n*)	80.3 (143)
Steroids, % (*n*)	50 (87)
Immunomodulators, % (*n*)	38.5 (67)
Anti‐TNF therapy, % (*n*)	21.8 (38)
Smoking history, % (*n*)	20.1 (35)
Surgical history for	
Perianal disease, % (* n*)	33.9 (59)
Intestine, % (*n*)	25.3 (44)

BMI, body mass index; FMT, faecal microbiota transplantation; HBI, Harvey–Bradshaw Index; HS‐CRP, hypersensitive C‐reactive protein; IQR, interquartile range; TNF, tumour necrosis factor.

### Clinical efficacy post‐FMT

**Figure 1:**
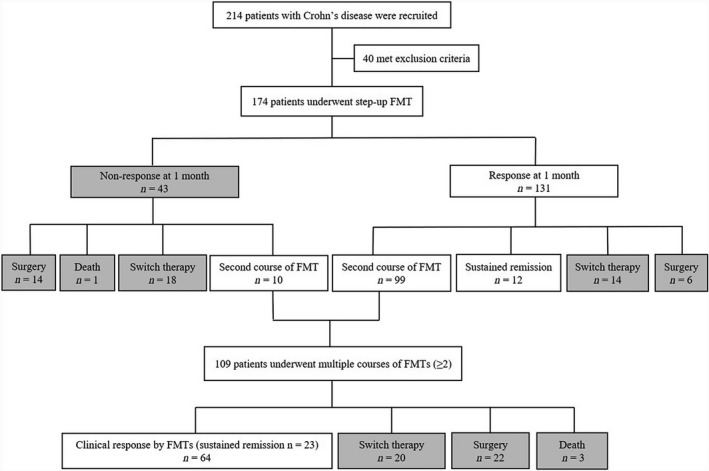
Outcomes of step‐up FMT strategy in CD patients.

As illustrated in Fig. 1, 75.3% (131/174) of patients achieved clinical response at 1 month after FMT. Of them, 9.2% (12/131) of patients achieved sustained remission after a single FMT, 75.6% (99/131) of patients underwent a second course of FMT, while 10.7% (14/131) of patients switched therapy due to loss of response during the follow‐up. In total, 109 patients underwent multiple courses of FMT during the follow‐up. Of them, 58.7% (64/109) of patients achieved clinical response with FMTs and 21.1% (23/109) of patients achieved sustained clinical remission with FMTs till the end of follow‐up. The overall median frequency of FMT courses was 3.5 (IQR, 2–5). The median time between the first and second course of FMT was 123 days (IQR, 97–251). By the final follow‐up, 43.7% (76/174) of patients achieved clinical response and 20.1% (35/174) of patients achieved sustained clinical remission based on the step‐up FMT strategy.

As summarized in Table S2, there were no significant differences in the clinical efficacy between groups that were divided according to the genetic background, mean age of donors (14 years) and status of microbiota. Frozen FMT decreased the rate of clinical response at 1 month by 11.3% when compared with fresh FMT. Similar findings were reported in our previous report (Cui *et al*., 2015a).

In univariate analysis, *P*‐value less than 0.150 was attributed to the disease duration, disease activity, disease location, disease behaviour, previous anti‐TNF therapy prior to first FMT and age of donor. Further multivariate analysis revealed that disease duration > 5 years (odds ratio, OR: 0.45; 95% confidence interval CI: 0.22–0.92; *P* = 0.029) and Harvey–Bradshaw Index (HBI) ≥ 8 (OR: 0.45; 95% CI: 0.21–0.95, *P* = 0.036) were independent predictors of poor response to FMT (Table S2). Patients with penetrating disease tended to have lower possibility to achieve clinical response when compared with those with non‐stricture non‐penetrating disease. Previous use of anti‐TNF therapy decreased the rate of clinical response at 1 month by 18.9% when compared with patients without previous use of anti‐TNF therapy. However, there were no significant statistical differences in the multivariate analysis (Table S2).

### Improvement of each target post‐FMT

**Figure 2:**
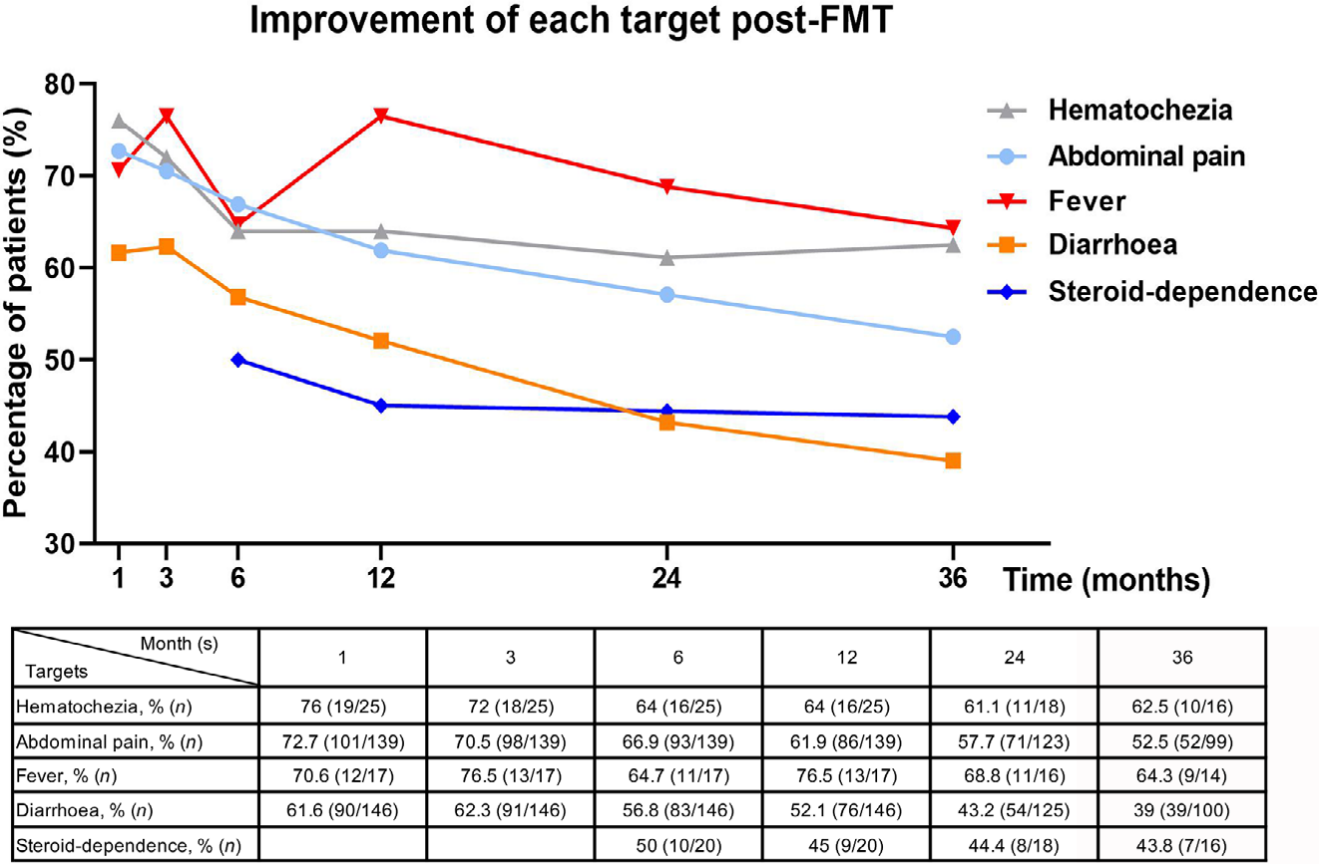
Improvement of each target at serial assessment time point post‐FMT. Percentage of patients who achieved improvement in abdominal pain, diarrhoea, hematochezia, fever at 1, 3, 6, 12, 24, 36 months post‐FMT and steroid‐dependence at 6, 12, 24, 36 months post‐FMT. The original data were shown as the table below, including specific number and percentage of patients who achieved improvement in each target, number of patients with follow‐up data at serial assessment time point.

In the study, 79.9% (139/174), 83.9% (146/174), 14.4% (25/174), 9.8% (17/174) and 11.5% (20/174) of patients complained of abdominal pain, diarrhoea, hematochezia, fever and steroid‐dependence before FMT respectively. The median total score of targets before FMT was 2 (range, 1–4). The total score decreased significantly at 1 month (*P* < 0.001), 3 months (*P* < 0.001), 6 months (*P* < 0.001), 12 months (*P* < 0.001), 24 months (*P* < 0.001) and 36 months (*P* < 0.001) after FMT. The changes in the percentage of patients who had shown improvement in each target are depicted in Fig. 2. Active perianal fistula and enterocutaneous fistula were not listed because of the limited sample size. As illustrated in Fig. 2, 76% and 72.7% of patients achieved improvement in hematochezia and abdominal pain, respectively, at 1 month after FMT, which were the highest values amongst the five targets. The percentage of patients who had shown improvement in the target of fever was the third highest (70.6%) and varied during the follow‐up. Furthermore, 61.6% of patients had shown improvement in the target of diarrhoea at 1 month after FMT; this figure remained stable until 12 months after FMT. Lastly, 50% of steroid‐dependent patients were steroid‐free at 6 months after FMT.

**Figure 3:**
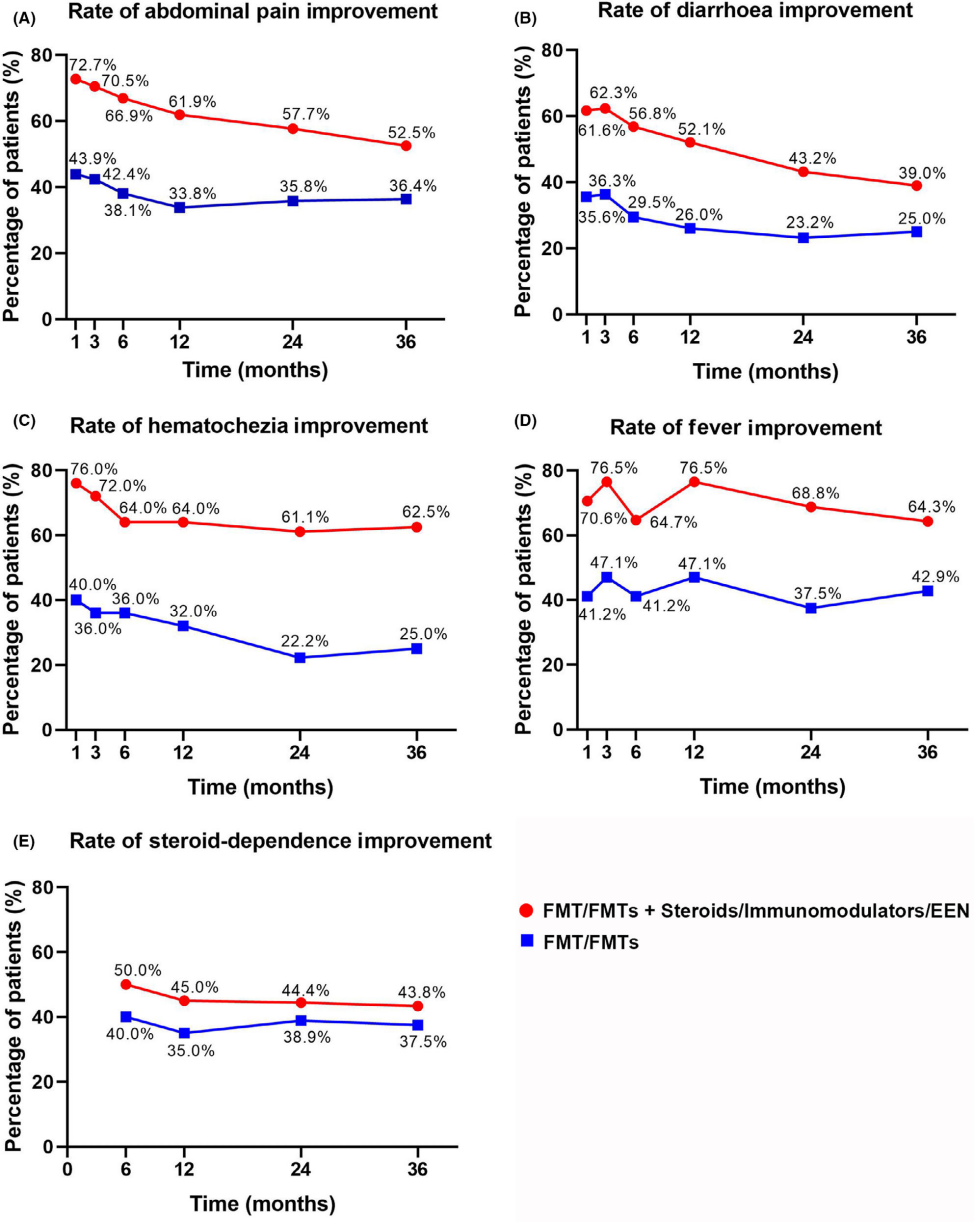
Improvement in each target in two groups divided by whether patients underwent step 3. EEN, exclusive enteral nutrition. Percentage of patients who achieved improvement in abdominal pain (A), diarrhoea (B), hematochezia (C), fever (D) and steroid‐dependence (E) in two groups divided by whether they received steroids, immunomodulators or EEN (step 3) based on single or multiple FMTs (step 1/2).

As illustrated in Fig. 3, the patients were divided into two groups based on whether they received steroids, immunomodulators and EEN (step 3) based on single and multiple FMTs (step 1 and 2 of step‐up strategy). Overall, 22.2%–47.1% of patients achieved improvements in the five targets based on single and multiple FMTs; however, a higher rate of improvement could be achieved with the use of step 3.

In terms of hematochezia before FMT, 92% (23/25) of patients complained of mild or moderate bleeding, whereas only two patients had severe intestinal bleeding that required blood transfusions. In one patient, the bleeding stopped within two weeks after FMT but recurred 5 months later, which required blood transfusion of four units. After the second FMT resulted in improvement in bleeding, 75 mg thalidomide per day was used and there was no recurrence of bleeding during the subsequent 9 months till the end of follow‐up. In another patient with intestinal bleeding and steroid‐dependence, there was no response to FMT and the patient died within two weeks after FMT because of refractory bleeding. Of the 10 patients who achieved steroid‐free remission after FMT, eight achieved clinical remission after one FMT, and one achieved clinical remission after two FMTs. Additionally, one patient achieved short‐term improvement after FMT and the disease flared within 2 months; subsequently, another FMT was performed, which resulted in clinical remission without relapse for 13 months. Five patients suffered from enterocutaneous fistula before FMT and the fistula closed in 4/5 (80%) of them within 1 month after FMT. Of these four patients, one experienced relapse 14 months after FMT and died of serious pulmonary infection and intestinal haemorrhage, while three did not experience relapse till the last follow‐up. Six patients complained of active perianal fistula before FMT; of them, 33.3% (2/6) achieved improvement, which was maintained until the last follow‐up. Of the remaining four patients without response, one maintained therapy with enteral nutrition and mesalazine, one switched to infliximab infusion, one underwent terminal ileostomy 7 months after FMT and one underwent perianal surgery after FMT. During the follow‐up, 24.1% (42/174) of all patients underwent intestinal surgery (resection or formation of a stoma) at a median of 23.5 months (range, 1–58) after FMT. In these patients, 40.5% (17/42) demonstrated non‐response at one month after FMT.

During the follow‐up, four patients died. One died within two weeks of FMT due to refractory intestinal bleeding, one died at one‐year post‐FMT from cardiopulmonary dysfunction, one died of serious pulmonary infection and intestinal haemorrhage at 20 months post‐FMT and one died of severe intestinal and enterocutaneous fistula at 54 months post‐FMT.

## Discussion

The principal finding of this study is that FMT resulted in improvement in clinical targets, especially in abdominal pain, hematochezia, fever and diarrhoea. To our knowledge, these data represent the largest cohort of patients with CD who underwent FMT and in whom the long‐term outcomes have been analysed for the first time.

Mucosal healing has been an important therapeutic target in the management of CD (Peyrin‐Biroulet *et al.*, 2015; Shah *et al.*, 2016). However, it remains difficult to achieve despite the extensive use of biologics and immunosuppressants (Cholapranee *et al.*, 2017; Noman *et al.*, 2017). Clinical targets, such as resolution of abdominal pain and normalization of bowel habits, are associated with patients’ quality of life.

The present study demonstrated that 72.7% of patients achieved improvement in CD‐related abdominal pain at 1 month after FMT and approximately half (52.5%) of the patients maintained the improvement three years after FMT. These findings are consistent with those of our previous report on FMT‐induced and sustained relief of CD‐related abdominal pain (Cui *et al*., 2015a). Another study reported that faecal microbiota composition was associated with the incidence of abdominal pain and its frequency, duration and intensity in the general population (Hadizadeh *et al.*, 2018). The aforementioned studies confirm that FMT contributes to the control of CD‐related abdominal pain.

Our findings revealed that 52.1% (76/146) of patients achieved improvement in diarrhoea within 12 months of FMT. Hematochezia, presenting as rectal bleeding or bloody diarrhoea, may be common in CD patients with colon involvement (Torres *et al.*, 2017). FMT has been reported to improve rectal bleeding in patients with ulcerative colitis (Paramsothy *et al.*, 2017). A total of 76% (19/25) of patients achieved improvement in hematochezia at 1 month after FMT. This is the first report of the efficacy of FMT against hematochezia in CD based on large series of cases.

The present results demonstrated that 50% (10/20) of steroid‐dependent patients achieved steroid‐free remission after FMT. Anti‐TNF therapy is recommended for steroid‐refractory or steroid‐intolerant patients with CD (Gomollon, *et al.*, 2017). However, 10%–40% of patients with inflammatory bowel disease failed to benefit from anti‐TNF therapy or lost response during use (Kennedy *et al.*, 2019). Therefore, FMT could be used as an alternative in steroid‐dependent patients and this might be related to microbiota regulating the host’s immune status and sensitivity to regular medications (Zhang *et al.*, 2018).

Fistulizing disease remains difficult to treat in clinical practice (Gionchetti *et al.*, 2017). The first successful treatment using FMT in patients with CD with serious infectious internal fistula in 2013 indicated the possibility of FMT for fistulizing CD as well (Zhang *et al.*, 2013). In the present study, we observed that enterocutaneous fistula closed in 80% (4/5) of the patients following FMT. Notably, all patients with enterocutaneous fistula in this study were treated with EEN for at least 1 month. EEN is considered to induce remission in CD (Grover *et al.*, 2014), and positive results were also observed in complicated CD (Yang *et al.*, 2017). A prospective observational study reported that 75% (3/4) of patients with enterocutaneous fistula experienced fistula closure after 12 weeks of EEN (Yang *et al.*, 2017). However, EEN reduces bacterial diversity and may increase microbial dysbiosis, which are intrinsic to its clinical efficacy (Quince *et al.*, 2015; MacLellan *et al.*, 2017). Therefore, FMT could reconstruct the gut microbiota and can be integrated with EEN to improve the closure rates of enterocutaneous fistula. Further studies are necessary to demonstrate whether FMT alone can help in the closure of enterocutaneous fistula.

The clinical efficacy of FMT reported in the present study was consistent with those reported in our previous studies (Cui *et al*., 2015a; He *et al.*, 2017; Wang *et al.*, 2018). In the multivariate analysis, long disease duration (> 5 years) and moderate or severe disease (HBI ≥ 8) were associated with poor response to FMT. This indicates that patients in the early stage of disease or with mild disease might show better response to FMT. Furthermore, patients may require multiple courses of FMT to consolidate its efficacy rather than a single treatment. The median duration between the first and second course of FMT was 123 days in this cohort. This was similar to that in our previous study in which the median time of maintaining clinical response to the first FMT was 125 days (Li *et al.*, 2019). We recommended 3 months post‐FMT as the ideal time for the second course of FMT in patients with CD (Li *et al.*, 2019). In terms of long‐term efficacy, it is noteworthy that until the end of follow‐up, nearly half (43.7%) of the patients achieved clinical response and one‐fifth (20.1%) of patients achieved sustained clinical remission after one or multiple courses of FMT. Although FMT alone did not frequently result in long‐term remission, it appears to have profound benefits in certain patients. The mechanism remains unknown, although increased α‐diversity and a shift towards the donor profile were reported in patients who benefited from the first FMT (Vaughn *et al.*, 2016; Gutin *et al.*, 2019; Li *et al.*, 2019). Additionally, it is worth investigating if FMT can alter the natural history of CD.

The safety of FMT in CD has been reported by our group recently (Wang *et al.*, 2018). In this study, no long‐term (> 1 m) FMT‐related AEs were observed. Four deaths during the follow‐up in this study were considered unrelated to FMT. No FMT‐related serious adverse events (SAEs) occurred during the follow‐up as well.

There are some limitations to this study. There was no control group for comparisons. Endoscopy findings and biomarkers were not included as targets. Quality of life was not analysed in the present study; however, evaluations of the quality of life and cost‐effectiveness were reported by our group previously (Zhang *et al.*, 2017). The current results based on washed microbiota preparation might be different from studies based on manual preparation. Additionally, microbial analysis was not performed in this study.

In conclusion, the findings of the present study indicated that it is important to understand the efficacy of FMT in CD against targeted therapeutics, especially abdominal pain, hematochezia, fever and diarrhoea. The present findings encourage us to re‐evaluate the therapeutic value of FMT in CD beyond the traditional evaluations that use HBI or Crohn’s Disease Activity Index.

## Experimental procedures

### Study design

A registered trial (NCT01793831) was performed between October 2012 and December 2017 for the use of FMT as treatment in CD. Patients with a documented diagnosis of CD with any therapeutic target, as defined in the subsequent paragraph, were included. The exclusion criteria were as follows: lost to follow‐up; change in the diagnosis during the follow‐up; severe comorbidities [e.g. *Clostridium difficile* infection (CDI), malignancy, cardiopulmonary failure and severe liver and kidney diseases]; and having undergone endoscopic dilation or perianal surgery within two weeks before FMT. The baseline patient characteristics were recorded, which included age, gender, weight, height, age at onset, age at diagnosis, disease duration, disease location, disease behaviour, HBI, history of intestinal and perianal surgery, history of smoking, history of drug use and combined medication therapy. Laboratory test results at baseline, such as blood haemoglobin and serum hypersensitive C‐reactive protein (HS‐CRP) and albumin were also recorded. Clinical outcomes, including clinical response, clinical remission, switching to other therapy, surgery or death were assessed by independent researchers at every medical visit or through telephone calls at 1 month after FMT and at the end of follow‐up. Researchers discussed ambiguous clinical assessments with the attending physicians of the patients. Improvement in each target was assessed based on medical records and telephone calls. The patients were followed up for at least 12 months. The primary outcome was the rate of improvement in each therapeutic target at 1, 3, 6, 12, 24 and 36 months after FMT. The secondary outcome was clinical response at 1 month after FMT.

### Definition of therapeutic targets

Seven targets were assessed and recorded as 1 (yes) or 0 (no) before FMT and during the follow‐up. These targets included abdominal pain, diarrhoea, hematochezia, fever, steroid‐dependence, enterocutaneous fistula and active perianal fistula. Steroid‐dependence was assessed at 6, 12, 24 and 36 months post‐FMT while other targets were assessed at 1, 3, 6, 12, 24 and 36 months post‐FMT. The total score of the targets was calculated by combining the score of each item. The target score was defined as 0 (no) for improvement in more than 80% of the duration between two serial time points. The detailed definitions are listed in Table 2. If patients underwent surgery or switched therapies after getting discharged from the hospital, the score was calculated as 1 during that period.

**Table 2:** Definition of each target and scoring method.

Target	Scoring method	
	1	0
Abdominal pain	Baseline: Mild, moderate or severe pain	(a) No abdominal pain; (b) degree of pain decreased from severe to mild
	Post‐FMT: Degree of pain from severe to moderate, from moderate to mild, unchanged or aggravated compared to baseline	
Diarrhoea	Baseline: Liquid stool	(a) No liquid stool; (b) frequency of liquid stool decreased from 2 to 1,> 2 to 0 ~ 2; (3) frequency of liquid stool decreased by 80% if > 10
	Post‐FMT: Frequency of liquid stool unchanged or increased compared to baseline	
Hematochezia	Blood in the stool	No blood in the stool
Fever	Fever (≥37.3 degree centigrade)	No fever (<37.3 degree centigrade)
Steroid‐dependence	(a) Relapse within 3 months of stopping steroids; (b) unable to reduce steroids below the equivalent of prednisone 10 mg day^−1^ within 3 months of starting steroids, without recurrent active disease	(a) Clinical remission without use of steroids; (b) steroids ceased for more than 3 months without relapse
Enterocutaneous fistula	Fistula drainage	(a) Closure of fistula; (b) no fistula drainage
Active perianal fistula	Moderate or substantial mucous or purulent discharge	(a) Closure of fistula; (b) a reduction in the number of draining fistulas by 50% compared to baseline

Definition: 1 = With any target at baseline; or matching any criteria for more than 20% of time between two serial assessment time points; 0 = Without target at baseline; or matching any criteria for more than 80% of time between two serial assessment time points.

### Step‐up FMT strategy

The step‐up FMT strategy (Cui *et al*., 2015b; Cui *et al.*, 2016; Ding *et al.*, 2019) consists of three steps as detailed in our previous reports (Fig. S1) with the following basic outline: step 1: single FMT; step 2: ≥ 2 FMTs; and step 3: FMT(s) followed by steroids, immunomodulators, or EEN. Patients would undergo step 2 within approximately one week if they did not respond or had inadequate response to the first FMT. Patients who had inadequate response to step 1 or step 2 were treated with steroids that were tapered off after 2–4 weeks of full‐dose oral therapy (prednisone 0.75–1.0 mg kg^−1^ day^−1^). Azathioprine and thalidomide were possibly administered during the tapering of steroids or as maintenance therapy after FMT. Patients were administered EEN for at least 1 month if they had malnutrition, severe stricture or intestinal fistula. Pre‐antibiotic treatment was not used before FMT for improving the efficacy.

### Efficacy and safety assessment

Clinical remission was defined as HBI ≤ 4, while clinical improvement was defined as a decrease of HBI score > 3. Clinical response included clinical improvement and clinical remission. Adverse events (AEs) were recorded during FMT and throughout the follow‐up period. The Common Terminology Criteria for Adverse Events (version 5.0) were used to describe the intensity and relationship of the adverse events with FMT. SAEs were defined as any death, life‐threatening experience, unplanned hospitalization or other important medical events (Kelly *et al.*, 2014; Wang *et al.*, 2018; Ding *et al.*, 2019).

### Donors and FMT procedure

Donors were screened according to our previously reported criteria (Ding *et al.*, 2019). Healthy donors aged from 5–24 years were selected from patients’ relatives or friends, or from our universal stool bank (Chinese fmtBank) (Table S3). Faecal microbiota was enriched in the laboratory by manual methods before April 2014 (Cui *et al*., 2015a; Ding *et al.*, 2019). Since then, an automatic purification machine (GenFMTer, Nanjing, China) has been used (Cui *et al.*, 2016). The microbiota preparation includes microfiltration with GenFMTer and the following repeated centrifugation plus suspension with support from specific facilities, which can improve the standardization of the laboratory processes, avoid the technicians’ exposure to faecal matter and reduce FMT‐related AEs (Wang *et al.*, 2018; Ding *et al.*, 2019; Zhang *et al.*, 2020). This methodology used in our group and most centres in China was recently coined as washed microbiota transplantation (WMT), which is dependent on the automatic facilities and washing process in a laboratory room (Zhang *et al.*, 2020). We adopted the one‐hour FMT protocol in which the duration between faeces defecation and infusion of the fresh bacterial material into the patient’s intestine is within one hour (Cui *et al.*, 2016). The purified faecal microbiota suspension (~ 50 cm^3^ faecal microbiota and ~ 100 ml normal saline) (Cui *et al*., 2015a) was delivered into the mid‐gut through gastroscopic infusion under anaesthesia, nasojejunal tube and mid‐gut TET (Long *et al.*, 2018) or into the lower gut through colonic TET (Peng *et al.*, 2016). In order to prevent reflux of the microbiota liquid and inhibit the secretion of gastric acid in patients who underwent endoscopic delivery, they were administered intramuscular metoclopramide 10 mg and intravenous proton pump inhibitor (PPI) at least one hour before FMT (Cui *et al*., 2015a). PPI and metoclopramide were not administered to patients who underwent mid‐gut tubing delivery.

### Statistical analysis

The continuous variables were expressed as mean, median with IQR or range. Qualitative variables were described as percentage. The changes in total score of targets between pre and post‐FMT were analysed by Friedman test. The impact factors of FMT efficacy were identified by univariate analysis using chi‐square tests and multivariate logistic regression analysis using a backward LR method. All variables with *P* value < 0.150 in univariate analysis were included in the multivariate logistic regression analysis. A two‐tailed *P* value of less than 0.050 was considered significant. Statistical analysis was performed using IBM SPSS Statistics version 20.0 (SPSS Inc., Chicago, IL, USA).

### Ethic approval

All subjects gave their informed consent before they participated in the study. The study was conducted in accordance with the Declaration of Helsinki, and the protocol was approved by the Second Affiliated Hospital of Nanjing Medical University Institutional Review Board (2012KY015).

## Conflicts of interest

Faming Zhang invented the concept of GenFMTer and transendoscopic enteral tubing and devices related to it. The other authors declare no conflict of interest.

## Author contributions

F.Z., B.C. and L.X. were involved in the study design and patient management. L.X., X.D., Q.L., X.W., M.D., C.L. and Z.H completed data collection. L.X. analysed the data and draw the manuscript. All authors reviewed and revised the manuscript and approved the final version of the manuscript.

## Supporting information


**Fig. S1**. The step‐up FMT strategy.

**Table S1**. FMT‐related donor, preparation, status and delivery route in the present study.
**Table S2**. Impact factors of response to FMT at 1 month.
**Table S3**. Criteria for donor screening.
