# Supplementary figures and images for: Efficacy of faecal microbiota transplantation in Crohn’s disease: a new target treatment?
Source: Microb Biotechnol. 2020 Jan 20;13(3):760–9. doi: 10.1111/1751-7915.13536 (PMC7111085; doi:10.1111/1751-7915.13536)

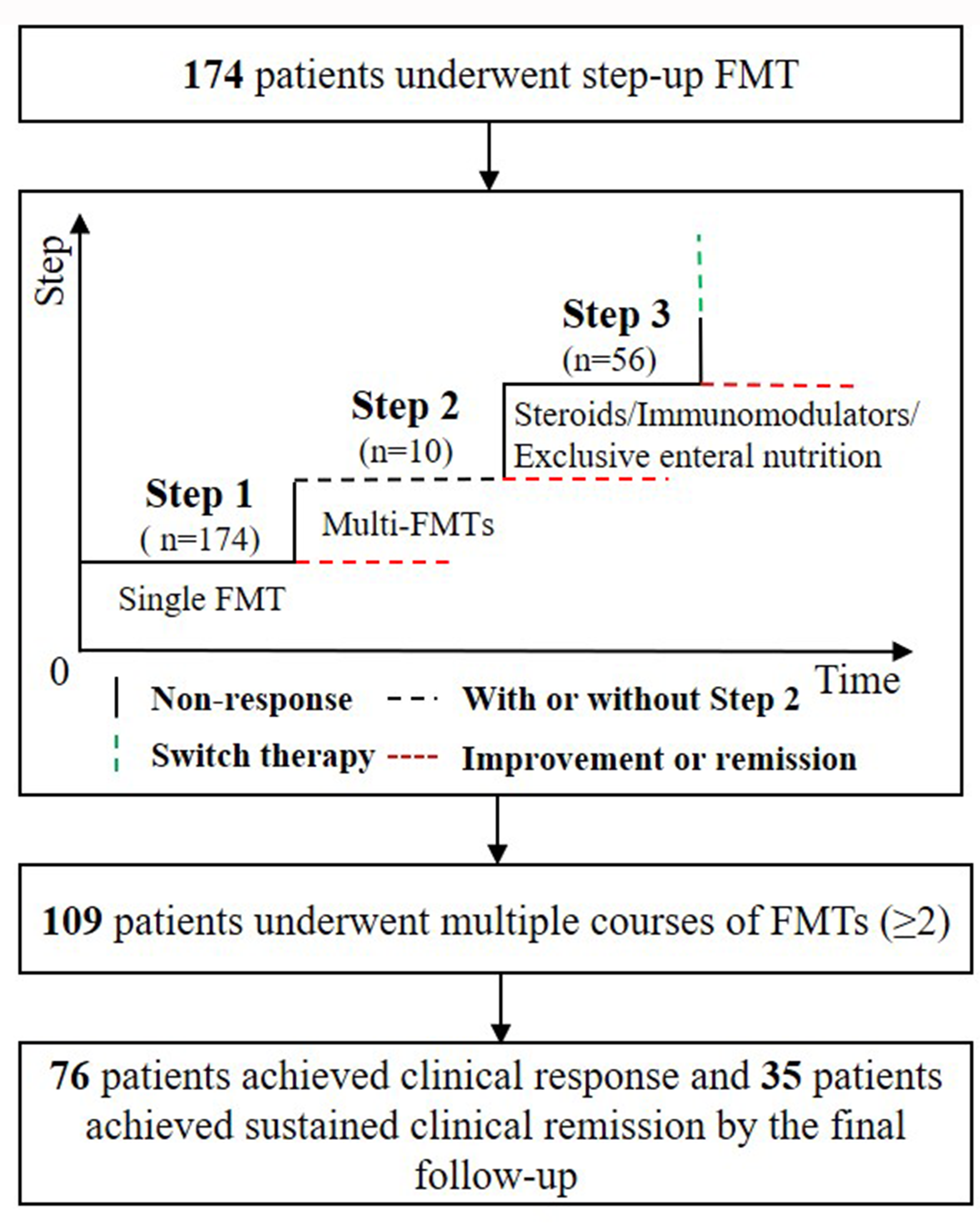

Supplement: Supplementary file 1 — Fig. S1. The step‐up FMT strategy. [file MBT2-13-760-s001.tif]
