# Supplementary material for: Efficacy of faecal microbiota transplantation in Crohn’s disease: a new target treatment?
Source: Microb Biotechnol. 2020 Jan 20;13(3):760–9. doi: 10.1111/1751-7915.13536 (PMC7111085; doi:10.1111/1751-7915.13536)
Supplement: Supplementary file 2 — Table S1. FMT‐related donor, preparation, status and delivery route in the present study. Table S2. Impact factors of response to FMT at 1 month. Table S3. Criteria for donor screening. [file MBT2-13-760-s002.docx]

**Efficacy of fecal microbiota transplantation in Crohn’s disease: A new target treatment?**

**Supplementary files**

Figure S1. The step-up FMT strategy

Table S1. FMT-related donor, preparation, status and delivery route in the present study

Table S2. Impact factors of response to FMT at 1 month

Table S3. Criteria for donor screening

**Figure S1.** The step-up FMT strategy

**Table S1.** FMT-related donor, preparation, status and delivery routes

| Items | Results |
| --- | --- |
| Donor  Total number  Age, mean±SD  Sex, male % (n)  One donor to ≥ 2 recipients, % (n) | 59  14.4±4.5  47.5 (28)  50.8 (30) |
| Genetic background, yes (%) | 14 (23.7) |
| Microbiota preparation methods |  |
| Manual, n (%) | 54 (31.0) |
| Automatic, n (%) | 120 (69.0) |
| Status of microbiota |  |
| Fresh, n (%) | 129 (74.1) |
| Frozen, n (%) | 17 (9.8) |
| Both, n (%) | 28 (16.1) |
| Delivery route |  |
| Mid-gut | 173 (99.4) |
| Colonic TET | 1 (0.6) |

After we had confirmed that the frozen FMT decreased the rate of clinical response when compared with the fresh FMT in CD in our earlier phase, the fresh FMT has become our recommended practice to CD patients.

**Table S2.** Impact factors of response to FMT at 1 month

| Items | Subgroups | Clinical response  % (n) | Univariate  P value | Multivariate | |
| --- | --- | --- | --- | --- | --- |
|  |  |  |  | OR (95% CI) | P value |
| Sex | Male  Female | 74.8% (89/119)  76.4% (42/55) | 0.823 | - | - |
| Age at first FMT (years) | ≤33 | 78.3% (72/92) | 0.335 | - | - |
|  | >33 | 72.0% (59/82) |  |  |  |
| Age at onset (years) | ≤25 | 75.0% (66/88) | 0.929 | - | - |
|  | >25 | 75.6% (65/86) |  |  |  |
| Disease duration (years) | ≤5  >5 | 82.2% (83/101)  65.8% (48/73) | 0.013 | 0.447 (0.217-0.920) | 0.029 |
| Moderate or severe disease (HBI ≥ 8) | Yes  No | 66.6% (60/90)  84.5% (71/84) | 0.006 | 0.450 (0.213-0.950) | 0.036 |
| Age at diagnosis | A1  A2  A3 | 81.5% (22/27)  74.5% (82/110)  73.0% (27/37) | 0.706 | - | - |
| Disease location | L1 | 69.0% (20/29) | 0.138 | Not retained | Not retained |
|  | L2 | 62.1% (18/29) |  |  |  |
|  | L3 | 81.2% (82/101) |  |  |  |
|  | L4 | 73.3% (11/15) |  |  |  |
| Disease behavior | B1 | 77.6% (59/76) | 0.100 | Not retained | Not retained |
|  | B2 | 79.4% (54/68) |  |  |  |
|  | B3 | 60% (18/30) |  |  |  |
| Intestinal surgery prior to first FMT | Yes  No | 72.7% (32/44)  76.2% (99/130) | 0.649 | - | - |
| Previous anti-TNF therapy prior to first FMT | Yes  No | 60.5% (23/38)  79.4% (108/136) | 0.017 | 0.481 (0.216-1.069) | 0.072 |
| Malnutrition (BMI<18.5) | Yes  No | 73.5% (86/117)  79% (45/57) | 0.435 | - | - |
| Perianal disease before FMT | Yes  No | 72.4% (21/29)  75.9% (110/145) | 0.694 | - | - |
| Smoking history | Yes  No | 80% (28/35)  74.1% (103/139) | 0.470 | - | - |
| Elevated CRP at baseline (>10mg/L) | Yes  No | 74.8% (89/119)  76.4% (42/55) | 0.823 | - | - |
| Anemia | Yes  No | 74.6% (88/118)  76.8% (43/56) | 0.752 | - | - |
| Hypoalbuminemia (albumin<3g/dl) | Yes  No | 83.3% (20/24)  74% (111/150) | 0.325 | - | - |
| Donor |  |  |  |  |  |
| Genetic background | Yes | 85.7% (12/14) | 0.522 | - | - |
|  | No | 74.4% (119/160) |  |  |  |
| Age (years) | ≤14 | 67.8% (40/59) | 0.101 | Not retained - | Not retained |
|  | >14 | 79.1% (91/115) |  |  |  |
| Status of microbiota | Fresh | 76.0% (98/129) | 0.560 | - | - |
|  | Frozen | 64.7% (11/17) |  |  |  |
|  | Both | 78.6% (22/28) |  |  |  |

FMT, fecal microbiota transplantation; HBI, Harvey Bradshaw Index; CRP, C-reactive protein; BMI, body mass index; TNF,

tumor necrosis factor; OR, odds ratio; Anemia: hemoglobin<13g/dl in male and <12g/dl in female and children.

**Table S3.** Criteria for donor screening

| FMT donor screening using eight-dimension criteria: age, physiology, pathology, psychology, veracity, time, living environment and recipients.  The protocol is briefly shown as follows. |
| --- |
| Inclusion criteria: (1) Donors age range 6 to 24 years old; (2) Physiologic criteria Body growth, body mass index, sleep quality, daily habits, diet, physical exercise and regular bowel habits should all be within normal value ranges. |
| Exclusion criteria: (1) Pathological criteria Antibiotic usage within three months, history of asymptomatic infection, tattoos or body piercing, incarceration or history of incarceration, allergies, immunological abnormalities, abnormal bowel habits in the previous year, infection with suspected or definite pathogen in the previous year, high-risk sexual behavior, illicit drug use, confirmed genetic risk factors, family members with diabetes, cancer, IBD and immune system diseases, bacterial infections, hepatitis viruses, human immunodeficiency virus, cytomegalovirus, togavirus, Epstein-Barr virus or other infectious diseases. (2) Psychological criteria A history of psychiatric and psychological disorders, suspected psychological abnormality and behavior abnormality. (3) Veracity Persons judged to have questionable integrity. (4) Living environment Living in geographic extremes (regions of high altitude, high temperature, alpine, cold, high. humidity, severely polluted areas, and saline-alkaline areas.); exposure to epidemic area within the past 3 months |
